# Supplementary material for: Humanized β2 Integrin-Expressing Hoxb8 Cells Serve as Model to Study Integrin Activation
Source: Cells. 2022 May 3;11(9):1532. doi: 10.3390/cells11091532 (PMC9102476; doi:10.3390/cells11091532)
Supplement: Supplementary file 1 [file cells-11-01532-s001.zip › cells-1634332-supplementary.pdf]

Figure S1

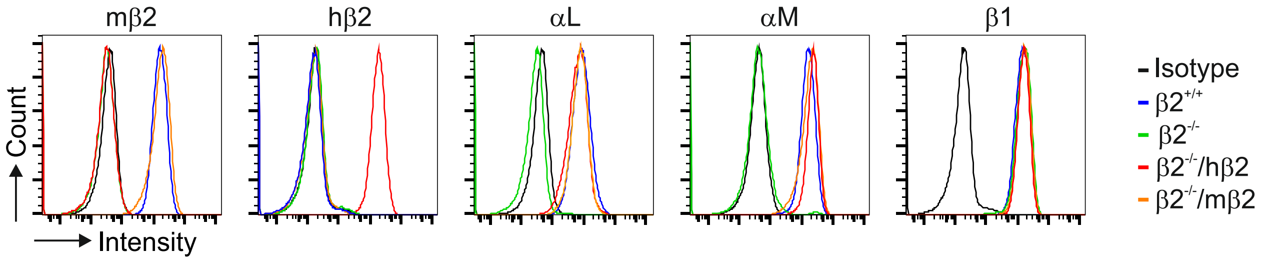

Figure S1. Expression of human integrin β2 in mouse integrin β2 knockout macrophages restores expression of corresponding integrin α-subunits. FACS analyses of the surface expression of mouse and human integrin β2, integrin αL, integrin αM and integrin β1 on macrophages differentiated from control ($β2^{+/+}$), integrin β2 ko ($β2^{-/-}$) and human or mouse integrin β2 expressing integrin β2 ko ($β2^{-/-}$/hβ2 and $β2^{-/-}$/mβ2) Hoxb8 FL cells.

Figure S2

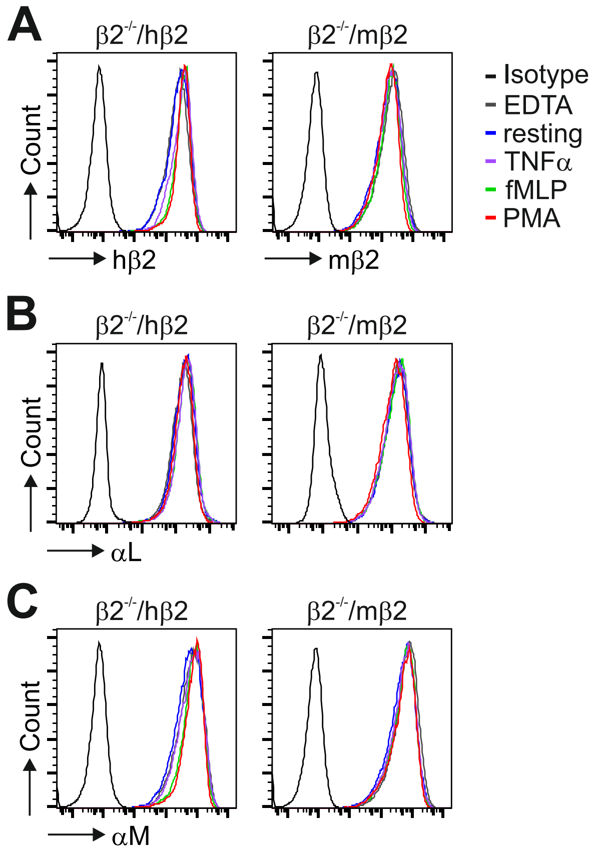

Figure S2. Surface expression of integrin subunits β2, αL and αM remains constant on activated neutrophil-like cells differentiated from human or mouse integrin β2 expressing integrin β2 ko ($\beta2^{-/-}$/hβ2 and $\beta2^{-/-}$/mβ2) Hoxb8 FL cells. (A+B) Surface expression levels of human/mouse integrin β2 (A), integrin αL (B) and integrin αM (C) on neutrophil-like integrin $\beta2^{-/-}$ cells retrovirally transduced with either human or mouse integrin β2, untreated or treated with EDTA, TNFα, fMLP or PMA.

Key Resources Table

Reagent type (species) or resource	Designation	Source or reference	Identifiers	Additional information
genetic reagent *(M.musculus)*	*ITGB2*$^{-/-}$	PMID: 9653089	RRID:MGI:2651618	
antibody	anti-CD11b-APC	Biolegend	Cat. #: 101211; RRID: AB_312794	FACS (1:2,000)
antibody	anti-CD11b-eF450	eBioscience	Cat. #: 48-0112-82; RRID: AB_1582236	FACS (1:300)
antibody	anti-CD16/CD32	BD Pharmingen	Cat. #: 553142; RRID: AB_394657	FACS (1:400)
antibody	anti-CXCR2 Alexa647	Biolegend	Cat. #: 149305; RRID: AB_2565693	FACS (1:200)
antibody	anti-p44/42 MAPK	Cell Signaling Technology	Cat. #: 4695, RRID:AB_390779	WB (1:5,000)
antibody	anti-phospho-p44/42 MAPK	Cell Signaling Technology	Cat. #: 4370, RRID:AB_2315112	WB (1:5,000)
antibody	mouse anti-GAPDH	Merck Millipore	Cat. #: CB1001; RRID: AB_2107426	WB (1:20.000)
antibody	anti-Gr-1-FITC	eBioscience	Cat. #: 11-5931-82; RRID: AB_465314	FACS (1:200)
antibody	anti-Gr-1-eFlour450	eBioscience	Cat. #: 48-5931-82; RRID: AB_1548788	FACS (1:200)
antibody	Peroxidase AffiniPure goat anti-mouse IgG (H+L)	Jackson Immunoresearch	Cat. #: 115-035-003; RRID: AB_10015289	WB (1:15.000)
antibody	Peroxidase AffiniPure goat anti-rabbit IgG (H+L)	Jackson Immunoresearch	Cat. #: 111-035-045; RRID: AB_2337938	WB (1:15.000)
antibody	anti-Integrin αL-PE	BD Pharmingen	Cat. #: 553121; RRID: AB_394637	FACS (1:200)
antibody	anti-Integrin β1-PE	BD Pharmingen	Cat. #: 562153; RRID: AB_10896298	FACS (1:200)
antibody	anti-Integrin β2-FITC	BD Pharmingen	Cat. #: 553292; AB_394761	FACS (1:200)
antibody	anti-Integrin β2-APC	BD Pharmingen	Cat. #: 562828; RRID: AB_2737823	FACS (1:200)
antibody	anti-Integrin β2-BV421 (mab24)	Biolegend	Cat. #: 363407; RRID: AB_2716067	FACS (1:100)
antibody	anti-Integrin β2 (KIM127)	Invivo Biotech Services GmbH	Clone name:ATCC-CRL-2838, batch number: AK2075/01.1	FACS (1:100)
antibody	anti-human Integrin β2-APC	Biolegend	Cat. #: 373405; RRID: AB_2716021	FACS (1:200)
antibody	rabbit anti-Kindlin-3	Markus Moser (MPI of Biochemistry)		WB (1:3,000)
antibody	mouse anti-paxillin	Thermo Fisher Scientific	Cat. #: 610051, RRID: AB_397463	WB (1:5,000)
antibody	rabbit anti-paxillin pY31	Thermo Fisher Scientific	Cat. #: 44-720G; RRID: AB_2533732	WB (1:1,000)
antibody	anti-PSGL-1 BV421	BD Pharmingen	Cat. #: 562807; RRID: AB_2737808	FACS (1:200)
antibody	anti-pY	Merck Millipore	Cat. #: 05-321, RRID:AB_309678	WB (1:1,000)
antibody	rabbit anti-Pyk2	Cell Signaling Technology	Cat. #: 3292, RRID: AB_2174097	WB (1:1,000)
antibody	rabbit anti-phospho-Pyk2 Y402	Cell Signaling Technology	Cat. #: 3291, RRID: AB_2300530	WB (1:1,000)
antibody	mouse anti-talin	Sigma-Aldrich	Cat. #: T3287; RRID: AB_477572	WB (1:20.000), IF (1:500)

peptide, recombinant protein	Truecut Cas9 Protein v2	Thermo Fisher scientific	Cat. #: A36498	
peptide, recombinant protein	Fibronectin Bovine plasma	Merck Millipore	Cat. #: F3506	5 µg/ml
peptide, recombinant protein	fMLP	Merck Millipore	Cat. #: 341632	10 µM
peptide, recombinant protein	recombinant mouse G-CSF	PeproTech	Cat. #: 250-05; Accession #: P09920	20 ng/ml
peptide, recombinant protein	recombinant human ICAM-1	R&D Systems	Cat. #: ADP4-200; Accession #: CAA30051	4 µg/ml
peptide, recombinant protein	recombinant mouse ICAM-1	stem cell technologies	Cat. #: ADP4-200; Accession #: CAA30052	3 µg/ml
peptide, recombinant protein	recombinant mouse IL-3	PeproTech	Cat. #: 213-13; Accession #: P01586	10 ng/ml
peptide, recombinant protein	recombinant mouse IL-6	PeproTech	Cat. #: 216-16; Accession #: P08505	20 ng/ml
peptide, recombinant protein	recombinant mouse KC	R&D Systems	Cat. #: 453-KC-010/CF ; Accession #: P12850	2 µg/ml (Flow chamber coating) 0.1 µg/ml (FACS)
peptide, recombinant protein	recombinant mouse P-selectin His-tag	R&D Systems	Cat. #: 10094-PS; Accession #: Q01102	5 µg/ml
peptide, recombinant protein	recombinant mouse TNFα	R&D Systems	Cat. #: 410-MT-025/CF; Accession #: P06804	0.2 µg/ml
peptide, recombinant protein	recombinant human VCAM-1	R&D Systems	Cat. #: ADP5-200; Accession #: P19320-1	4 µg/ml
chemical compound, drug	Alexa Fluor 647 Antibody labeling kit	Thermo Fisher Scientific	Cat. #: A20186	
chemical compound, drug	Ficoll-Paque Premium 1.084	GE-Healthcare	Cat. #: 17-5446-02	
chemical compound, drug	PMA	Merck Millipore	Cat. #: 524400	100 ng/ml
chemical compound, drug	Lipofectamine	Thermo Fisher Scientific	Cat. #: 18324012	1:1.000
chemical compound, drug	Lipofectamine 2000	Thermo Fisher Scientific	Cat. #: 11668019	
software, algorithm	FlowJo	BD Biosciences	RRID:SCR_008520	
software, algorithm	ImageJ	National Institute of Health	RRID:SCR_003070	
other	Glass Bottom Microwell Dishes	MatTek Corporation	Cat. #: 80661	
other	ibidi slides VI 0.1 uncoated	ibidi	Cat. #: P35G-1.5-20-C	
